# Celecoxib Alleviates Memory Deficits by Downregulation of COX-2 Expression and Upregulation of the BDNF-TrkB Signaling Pathway in a Diabetic Rat Model

**DOI:** 10.1007/s12031-017-0922-0

**Published:** 2017-05-02

**Authors:** Ying Yang, Ling Gao

**Affiliations:** Xiangyang Central Hospital, Affiliated Hospital of Hubei University of Arts and Science, No. 136, Jingzhou Street, Xiangcheng District, Xiangyang City, Hubei 441021 China

**Keywords:** Diabetes, Celecoxib, Memory deficit, Long-term potentiation, COX-2, BDNF-TrkB signaling

## Abstract

Previous studies conveyed that diabetes causes learning and memory deficits. Data also suggest that celecoxib exerts an anti-hyperalgesic, anti-allodynic, and a plethora of other beneficial effects in diabetic rats. However, whether celecoxib could alleviate memory deficit in diabetic rat is unknown. In the present study, we aimed to examine the potential of celecoxib to counter memory deficits in diabetes. Experimental diabetes was induced by streptozotocin (STZ, 60 mg/kg) in male SD rats. Rats were divided into three groups (*n* = 16/group): normal control group injected with normal saline, diabetes group injected with STZ, and diabetes + celecoxib group in which diabetic rats were administered with celecoxib by gavage in drinking water (10 mg/kg) for 10 days in terms of which memory performance in animals was measured, hippocampal tissue harvested, and long-term potentiation assessed. Western blotting and immunohistochemical staining were performed to determine cyclooxygenase 2 (COX-2) expression in hippocampus. The results showed that a rat model of STZ-induced diabetes was successfully established and that celecoxib treatment significantly improved the associated nephropathy and inflammation. Moreover, spatial memory and hippocampal long-term potentiation (LTP) were impaired in diabetic model (*P* < 0.05). Interestingly, our data revealed that oral application of celecoxib reversed the memory deficit and hippocampal LTP in the diabetic rats. To understand the underlying mechanisms, the expression of some important pathways involved in memory impairment was determined. We found that brain-derived neurotrophic factor (BDNF) and phosphorylated tropomyosin-related kinase (p-TrkB) were decreased in diabetic rats but were effectively reversed by celecoxib treatment. As evidenced by western blotting and immunohistochemical staining, the expression of COX-2 in hippocampus was significantly upregulated in diabetic rat (*P* < 0.05) but inhibited by celecoxib treatment. The present findings provide novel data that celecoxib reverses memory deficits via probable downregulation of hippocampal COX-2 expression and upregulation of the BDNF-TrkB signaling pathway in a diabetic rat.

## Introduction

Diabetes mellitus is the most common metabolic disorder worldwide, and its incidence is increasing annually. It was estimated that 382 million people or 8.3% of adults worldwide have diabetes in 2014 (International Diabetes Federation Atlas Sixth edition). Diabetes and insulin resistance are associated with changes in the central nervous system (CNS) and development of cognitive and memory impairments. The rising prevalence of diabetes and its earlier onset indicate that diabetes-related cognitive and memory dysfunction may increase substantially in the near future, causing tremendous socioeconomic burdens. Detrimental effects on cognitive functioning, such as verbal and visual memory, verbal and numerical reasoning, verbal fluency, concentration, and attention, are seen in both type 1 and type 2 diabetes, especially in the elderly. Cognitive dysfunction affects life quality of diabetic patients and increases the risk of dementia (Elias et al. 1997; Ott et al. 1996). Therefore, discovery of drugs to treat memory impairment in diabetes is very fundamental.

Celecoxib exhibits anti-pyretic, anti-inflammatory, and analgesic activities attributed to the inhibition of prostaglandin synthesis. However, other mechanisms including endogenous opioids have been proposed for this drug. The effects of celecoxib in diabetic pain have been scarcely studied. Celecoxib ameliorates non-alcoholic steatohepatitis in type 2 diabetic rats via suppression of the non-canonical Wnt signaling pathway expression (Tian et al. 2014). Celecoxib protects type 2 diabetes rats against non-alcoholic steatohepatitis probably via modulating the expression of PPAR gamma and NF-kappa B (Tian et al. 2016). Celecoxib reduces hyperalgesia and tactile allodynia in diabetic rats (Juarez-Rojop et al. 2015). The periocular administration of propranolol and celecoxib can significantly reduce ocular VEGF levels in a diabetic mouse model (Nassiri et al. 2016). Celecoxib exhibited potential anti-depressant-like effect in depression associated with obesity, which to some extent is mediated by reversing the altered plasma glucose in obese mice (Kurhe et al. 2014). However, the effect of celecoxib on memory deficits is largely unknown in diabetes. Therefore, the current study was designed to investigate the effects of celecoxib on memory ability in diabetic rat.

The mechanisms underlying memory deficit in diabetes are largely unclear. Learning and memory are related to many biological structures, and hippocampus and associated areas are important in memory formation (Hasselmo et al. 1996). Chronic hyperglycemia in rats was reported to produce a significant loss of cortical neurons, which may impair cognition (Redish and Touretzky 1997). In addition, neurotransmitter systems, such as gamma-aminobutyric acid and cholinergic systems, are involved in learning and memory (Everitt and Robbins 1997). Animal studies indicate that chronic hyperglycemia induced by streptozotocin (STZ) decreases the synthesis and release of acetylcholine in rat brain (Welsh and Wecker 1991). Cyclooxygenase 2 (COX-2) is expressed under inflammatory conditions, and its product prostaglandin E is an important inflammation mediator. A previous study demonstrated the importance of COX-2 activity in autoimmune destruction of beta cells, and inhibition of COX-2 can be protective against the development of diabetes mellitus. Histological analysis indicated that STZ-mediated destruction of beta cells was prevented by COX-2 inhibitor NS-398, and delayed administration of NS-398 at day 3 was also protective in this model (Tabatabaie et al. 2000). Meanwhile, enhanced nuclear factor-κB (NF-κB) activity was reported to impair vascular function by COX-2-dependent mechanisms in type 2 diabetic mice (Kassan et al. 2013). In addition, the inhibition of COX-2 was reported to be neuroprotective in different disease models. COX-2 inhibitor reduced inflammation and improved functional outcomes in a rat model of traumatic brain injury (Gopez et al. 2005). COX-2 inhibitor was also revealed to attenuate cytotoxicity of chronic neuroinflammation in basal forebrain cholinergic neurons of rats (Willard et al. 2000). The expression of COX-2 increased significantly in hippocampus in a rat model of global cerebral ischemia reperfusion injury, suggesting an important role of COX-2 inhibition in protecting cognitive impairments during ischemia and stroke (Yu et al. 2014). However, it is unclear whether COX-2 plays a role in the potential effect of celecoxib on cognitive dysfunction in diabetes.

Brain-derived neurotrophic factor (BDNF)-tyrosine receptor kinase B (TrkB) signal transduction pathway modulates synapse stability (Caldeira et al. 2007; Zhu et al. 2015a), GABAergic signaling (Henneberger et al. 2002), dendritogenesis (Yoshii and Constantine-Paton 2007; Zhu et al. 2015b), and neurogenesis (Bartkowska et al. 2007). Enhanced learning and memory was linked to BDNF/TrkB-signaling during multiple chronic stresses (Li et al. 2007). Interference of BDNF/TrkB-signaling was shown to impair long-term potentiation (LTP) in mouse hippocampus (Schildt et al. 2013). Moreover, decreased mRNA expression of BDNF was detected in the hippocampus of patients with mood disorders and schizophrenia (Reinhart et al. 2015; Thompson Ray et al. 2011). However, it is unclear whether BDNF/TrkB signaling plays a role in the potential effect of celecoxib on cognitive dysfunction in diabetes.

Thus, the purpose of this study was to assess the effects of celecoxib on memory impairment in diabetic rats and explore the involvement of COX-2 and BDNF/TrkB signaling in the underlying mechanisms.

## Methods

### Animals and Experimental Design

Sprague Dawley rats (SD rats; weight = 120 ± 10 g, male, 6 weeks) were obtained at China Medical University. All the rats used in this study were kept under a 12-h light–dark cycles at a constant temperature and humidity with free access to chow and water. The animal procedures were approved by the Animal Use and Care Committee of Xiangyang Central Hospital Affiliated to Hubei University of Arts and Science and were conducted following the protocols established by this institution. After 2-week acclimation, animals were randomly distributed into two groups for the establishment of diabetes model: normal control injected with normal saline (*n* = 16) and diabetic group (*n* = 16/group) injected with a single dose of STZ (Sigma Inc., St. Louis, MO; 60 mg/kg body weight) intraperitoneally. Blood glucose was measured weekly from tail vein using point of care blood glucose monitoring system (Accu-Chek Advantage, Roche Diagnostics, USA). The body weight was also measured every week. Four weeks following the STZ injections, rats with blood glucose levels of ≥16.7 mmol/L were considered diabetic. In order to determine the capacity of celecoxib to rescue from memory deficit, the STZ-induced diabetic rats were divided into two groups (*n* = 16 each) and administered with vehicle (saline, gavage, daily) as the diabetic group or celecoxib (20 mg/kg, gavage, daily; Pfizer, New York, NY) as the diabetic + celecoxib group for 10 days. At the end of the drug treatment, rats were anesthetized with chloral hydrate (3 mL/kg of body weight) and sacrificed by exsanguination.

#### Electrophysiological Experiments

Ten days after drug treatment, animals were submitted to ether anesthesia and quickly decapitated. Next, the skull was opened by dissection and the whole brain removed from it and placed in HEPES solution (NaCl = 130 mM, KCl = 4.9 mM, CaCl_2_·2H_2_O = 1.5 mM, MgSO_4_·7H_2_O = 0.3 mM, MgCl_2_·6H_2_O = 11 mM, KH_2_PO_4_ = 0.23 mM, Na_2_HPO_4_·2H_2_O = 0.775 mM, glucose = 5 mM, HEPS = 25 mM, NaHCO_3_ = 22 mM, pH = 7.3–7.4) at 0–4 °C for 2 min, under a 95% O_2_ and 5% CO_2_ environment. Next, the bilateral hemispheres were separated by a longitudinal incision along the sagittal suture and placed on ice with the cut surface facing the ice platform. Subsequently, a third of the parietal lobe was cut by placing the blade at an angle 50–70° relatively to the horizontal plane and glued on the vibrating slicer on the stage. After the fixation, the hippocampus was cut into slices of about 350-μm thickness. The cut hippocampal slices were quickly transferred to a continuous carbon-oxygen mixture (95% O_2_–5% CO_2_) with a wide-caliber glass pipette to maintain a constant temperature of 32 °C at an artificial cerebrospinal fluid perfusion speed (NaCl = 130 mM, KCl = 4.9 mM, CaCl_2_ = 1.5 mM, MgSO_4_ = 0.3 mM, MgCl_2_ = 11 mM, KH_2_PO_4_ = 0.23 mM, Na_2_HPO_4_ = 0.8 mM, glucose = 5 mM, HEPES = 25 mM, NaHCO_3_ = 22 mM, pH = 7.3–7.4) of 5 mL/min. The field potential was recorded and the excitatory postsynaptic potential (EPSP) at Schaffer Collateral-CA1 field was recorded 1 h after incubating brain slides in the water bath. LTP was achieved through theta burst stimulation (TBS; ten bursts of four pulses at 100 Hz, delivered at 5 Hz). Input, output, and pair-pulsed facilitation were also recorded.

#### Western Blot

Hippocampal tissue was harvested at day 10 after drug treatment. Proper amounts of RIPA were added to grind tissue and extract total protein. Then, protein samples were mixed with protein loading buffer at a ratio of 1:1 and the mixture boiled for 5 min. Next, samples were purified by SDS-PAGE and transferred to nitrocellulose membrane at 200 mA for 2 h and blocked with 5% nonfat dried milk on a shaker at room temperature for 3 h and washed with phosphate-buffered solution with Triton (PBST) for three times, 10 min each washing. Then, the membrane was incubated with primary antibodies (BDNF: Abcam, Cambridge, MA, 1:1000; P-TrkBz: Abcam, Cambridge, MA, 1:1000; GAPDH: Abcam, Cambridge, MA, 1:5000; COX-2: Abcam, Cambridge, MA, 1:1000) at 4 °C overnight and washed with PBST for three times. The membrane was then incubated with goat anti-rabbit secondary antibody (Invitrogen, Waltham, MA; 1:3000) at room temperature for 2 h and washed with PBST for three times. After enhancement with electrochemiluminescence (ECL) kit, films were exposed in a dark room and densitometric analysis performed using ImageJ.

#### Detection of COX-2 Activity

Hippocampal tissue was harvested at day 10 of drug treatment. COX Activity Assay Kit (Abcam Inc., Boston, MA, USA) was used to detect COX-2 activity in hippocampal tissue homogenate among control, diabetic, and diabetic + celecoxib groups following the manufacturer’s instructions.

#### Water Maze Testing

The Morris water maze experiments were performed using a circular metallic apparatus (50-cm high and diameter of 180 cm) filled with a 20 °C water. A 10 cm in diameter escape platform was put in the pool and immersed 1.5 cm beneath the water surface. At the beginning of the trial, the mice were allowed to swim in the pool (free of platform) for 5 min of acclimation with the maze. The test lasted for 5 days and was scheduled for a fixed period of time every day. At the beginning of the training, the platform was placed in the NW quadrant and the mouse was placed into the pool at any point from the four starting points of the wall. The free video recording system was used to record the time (escape latency) and swimming path of the rats in the platform; the rats were given four different starting points (different quadrants) into the water. After the rats found the platform (latency recorded as 120 s), they were taken out by the experimenter and put on the platform for 15 s resting before the next test. The average of the four incubation periods per day in rats was used as the learning achievement.

#### Blood Biochemical Analysis and Detection of Serum BDNF

Blood glucose, serum creatinine, blood urea nitrogen (BUN), tumor necrosis factor alpha (TNF-α), interleukin 6 (IL-6), C-reactive protein (CRP), and serum BDNF (R&D Systems Inc., Minneapolis, MN) were detected according to instructions of kits, respectively.

#### Statistical Analysis

Results were presented as mean ± SEM. Differences among groups were compared using one way analysis of variance (ANOVA), followed by Bonferroni post hoc testing for multiple comparisons. Differences between two groups were assessed by Student’s t test. *P* values of 0.05 or less were regarded significant.

## Results

### Celecoxib Treatment Decreased Blood Glucose Levels and Improved Renal Functions of Diabetic Rats

**Fig. 1:**
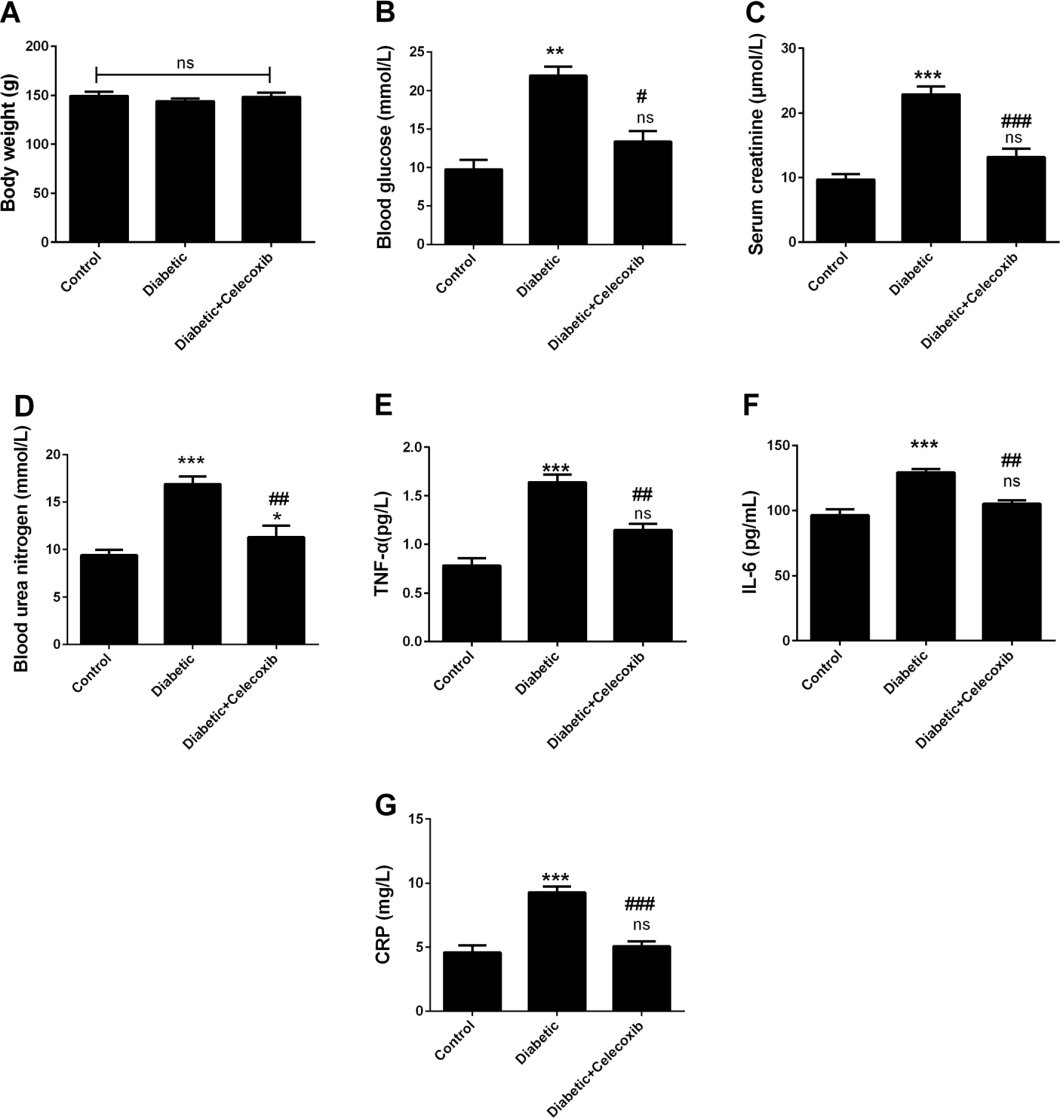
Celecoxib treatment decreased blood glucose levels and improved renal functions of diabetic rats. **a** Effects of STZ and celecoxib on body weight. Body weight of animals was similar among three groups. **b** Blood glucose, **c** creatinine, and **d** BUN increased in diabetic animals compared to control group. The increase in blood glucose, creatinine, and BUN was attenuated by celecoxib treatment. **e** TNF-α, **f** IL-6, and **g** CRP levels were detected by ELISA. Diabetic animals had increased concentration of TNF-α, IL-6, and CRP. The increase in TNF-α, IL-6, and CRP was attenuated by celecoxib treatment (mean ± SEM, *n* = 5 animals/group). *Three asterisks* indicate that *P* < 0.001 as compared to the control group. *One*, *two*, and *three number signs* indicate that *P* < 0.05, *P* < 0.01, and *P* < 0.001 as compared to diabetic group. *ns* no significance

In this investigation, we successfully established a rat model of STZ-induced diabetes with blood glucose concentration of ≥16.7 mmol/L. Neither STZ administration nor celecoxib treatment significantly affected rat body weight when compared to the control animals (Fig. 1a). The concentration of blood glucose in the STZ-induced diabetes group was significantly increased relatively to the control group (Fig. 1b). In the group of the diabetes animals treated with celecoxib, the levels of elevated blood glucose were remarkably (*P* < 0.01) reduced compared with the diabetic rats (Fig. 1b), thus implying that celecoxib is a potential attenuator of blood glucose concentration in the diabetic rats. To examine the renal injury and dysfunction induced by STZ injection and whether celecoxib could improve renal function of diabetic rats or not, indicators of renal function, creatinine, and BUN, were determined. As a result, each of the indicators was significantly elevated in diabetes group relatively to the control group (*P* < 0.01) (Fig. 1c, d). These data further indicated that the STZ-induced diabetes rat model was valid and the diabetic rats had suffered from renal dysfunction. More interestingly, with celecoxib treatment, these urinary indicators were markedly decreased compared with the diabetes group (*P* < 0.05). We also found that celecoxib treatment attenuated the increase of inflammatory markers in diabetic animals. Especially, the concentrations of TNF-α, IL-6, and CRP were increased in the diabetic animals (Fig. 1e–g). However, these increased levels of TNF-α, IL-6, and CRP were attenuated by celecoxib treatment, respectively (Fig. 1e–g). Thus, these observations indicated that STZ injection induced diabetes in rats and that celecoxib treatment significantly alleviated the associated nephropathy and inflammation.

### Celecoxib Improves Memory Deficit in the Diabetic Animals

**Fig. 2:**
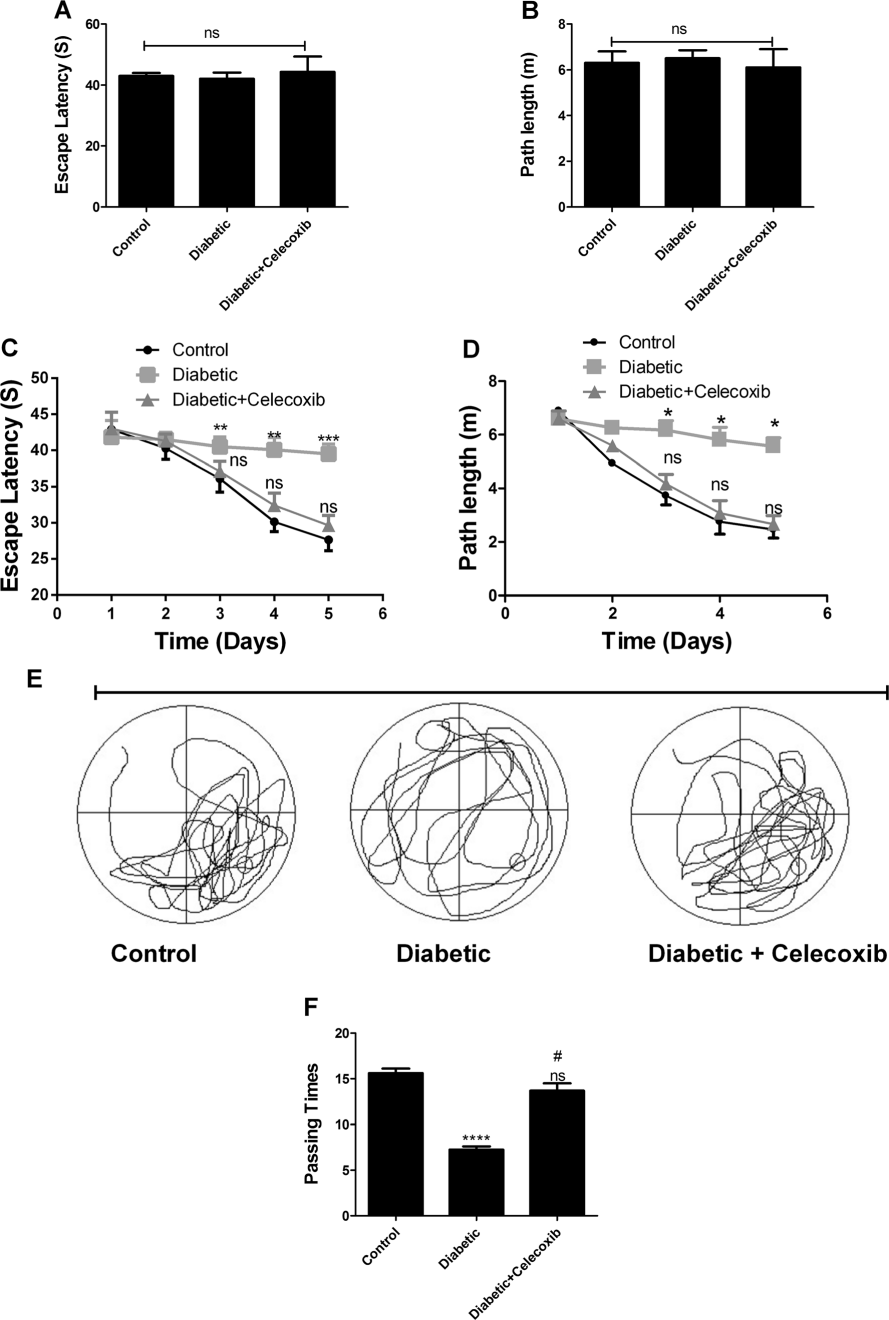
Celecoxib improves memory deficit in the diabetic animals. Water maze tests were used to detect learning memory of rats 10 days after drug treatment. **a** Escape latency in normal control group, diabetic model control group, and celecoxib treatment group during the visible platform test. **b** Path length in normal control group, diabetic model control group, and celecoxib treatment group during the visible platform test. **c** Escape latency in normal control group, diabetic model control group, and celecoxib treatment group during the hidden platform test. **d** Path length in normal control group, diabetic model control group, and celecoxib treatment group during the hidden platform test. **e** Swimming trajectory of rats captured by video camera in three groups. **f** Number of times animals in normal control group, diabetic model control group, and celecoxib treatment group visited the third quadrant the last day of the hidden platform test. *One*, *two*, *three*, and *four asterisks* indicate that *P* < 0.05, *P* < 0.01, *P* < 0.001, and *P* < 0.0001 as compared to the control group. *Number sign* indicates that *P* < 0.05 as compared to diabetic group. *ns* no significance

The rats were monitored during the whole process of drug treatment. Celecoxib administration did not influence body weight and outward manifestation of the animals. One day after the last administration, the effect of celecoxib on learning memory of rats was evaluated using the Morris water maze tests. First, by performing the visible platform tests, we found no significant difference among the control, diabetic, and diabetic + celecoxib groups in escape latency (Fig. 2a) and path length (Fig. 2b). These observations implied that rats in the three groups had comparable motor and visual abilities. Next, the hidden platform trials were performed over 5 days. The results indicated that there was a significant difference between the control group and the diabetic group in terms of escape latency (Fig. 2c) and path length (Fig. 2d). In addition, in the group of diabetic rats treated with celecoxib, the escape latency (Fig. 2c) and path length (Fig. 2d) were significantly decreased compared to the diabetic animals, suggesting that celecoxib treatment rats significantly performed better compared to the diabetic rats. There was no significant difference between the normal control group and the diabetic + celecoxib group. Figure 2e depicts swimming trace in the spatial probe test. On the last day, the probe trail experiments indicated an increased visit frequency of rats in the diabetic + celecoxib group into the third quadrant, where the hidden platform was formerly placed relatively to the diabetic rats with no significant difference when compared to control (Fig. 2f). These observations suggested that celecoxib improves memory deficits in diabetic rats.

### Celecoxib Improves Hippocampal LTP and Hippocampal Basal Synaptic Transmission in the Diabetic Animals

**Fig. 3:**
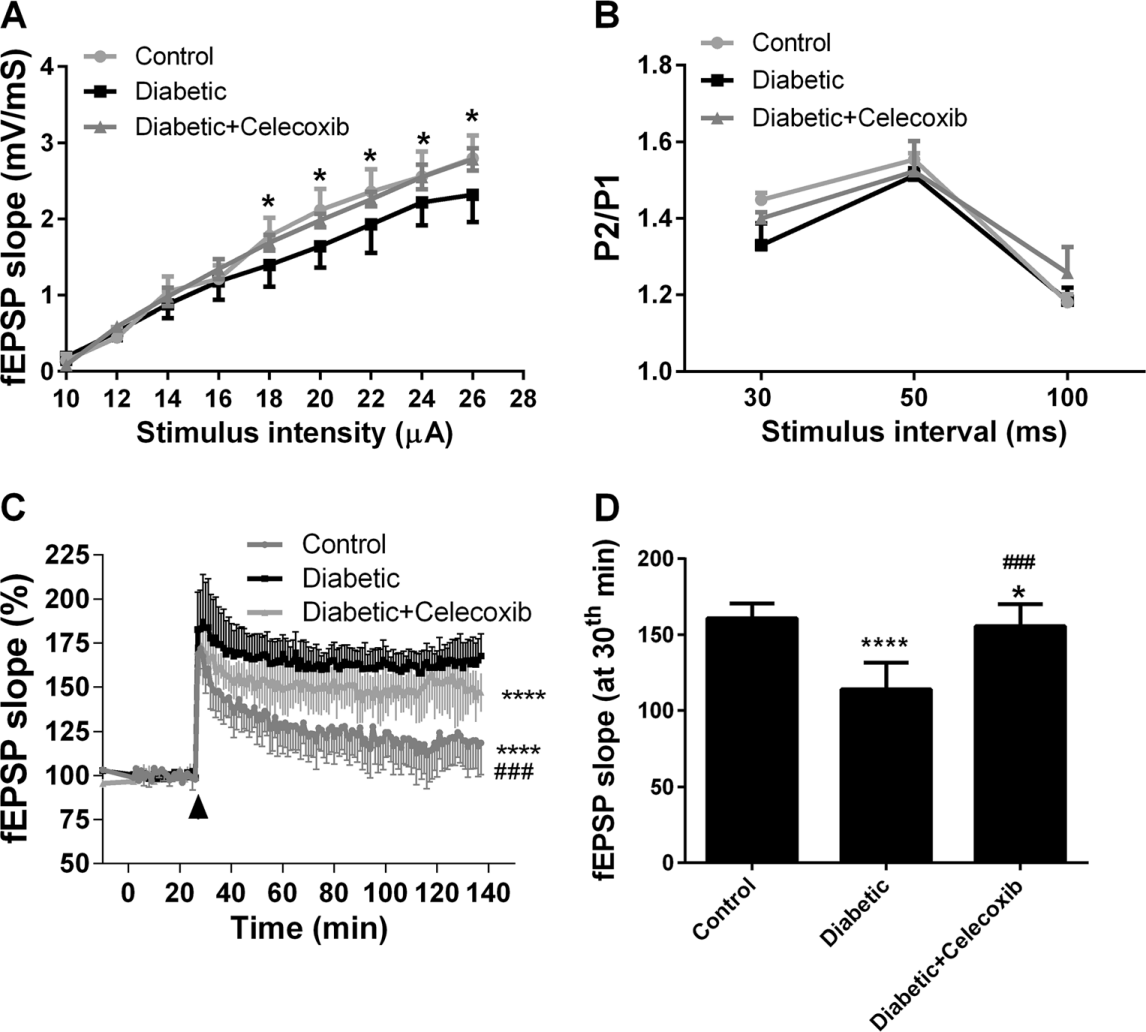
Celecoxib improves hippocampal long-term potentiation (LTP) and hippocampal basal synaptic transmission in the diabetic animals. EPSP at Schaffer Collateral-CA1 field was recorded. LTP was achieved through theta burst stimulation. Input, output, and pair-pulsed facilitation were recorded. **a** fEPSP slope at different electric current. **b** P2 to P1 ratio at different time points in three groups (mean ± SEM, *n* = 16 animals/group). **c** fEPSP slope after stimulation in three groups. **d** fEPSP slope at 30th min after stimulation in three groups. *One* and *four asterisks* indicate that *P* < 0.05 and *P* < 0.0001 as compared to the control group. *Three number signs* indicates that *P* < 0.001 as compared to diabetic group. *ns* no significance, *fEPSP* field excitatory postsynaptic potential, *LTP* long-term potentiation, *STZ* streptozotocin

The effects of celecoxib treatment on LTP and hippocampal basal synaptic transmission were measured in the rats at the last day of celecoxib administration. The fEPSPs at Schaffer collateral-CA1 synapses were measured. The input–output (stimulation–response) was apparently decreased in diabetic group but the input–output hippocampal Schaffer collateral-CA1 synapses was enhanced after celecoxib administration compared to the diabetic (Fig. 3a). There no significant difference among groups concerning the paired-pulse facilitation (Fig. 3b). TBS-induced LTP in diabetic group was significantly higher compared to control group (Fig. 3c). However, celecoxib treatment clearly decreased TBS-induced LTP when compared to the diabetic group (Fig. 3c). The LTD induced by low-frequency (fEPSP slope at 30th min) was significantly decreased in the diabetic group compared to control and diabetic + celecoxib groups (Fig. 3d). These results suggest that celecoxib treatment improved the hippocampal basal synaptic transmission and LTP.

### Celecoxib Improves the Damage in BDNF-TrkB Signaling Pathway in Hippocampus of the Diabetic Model

**Fig. 4:**
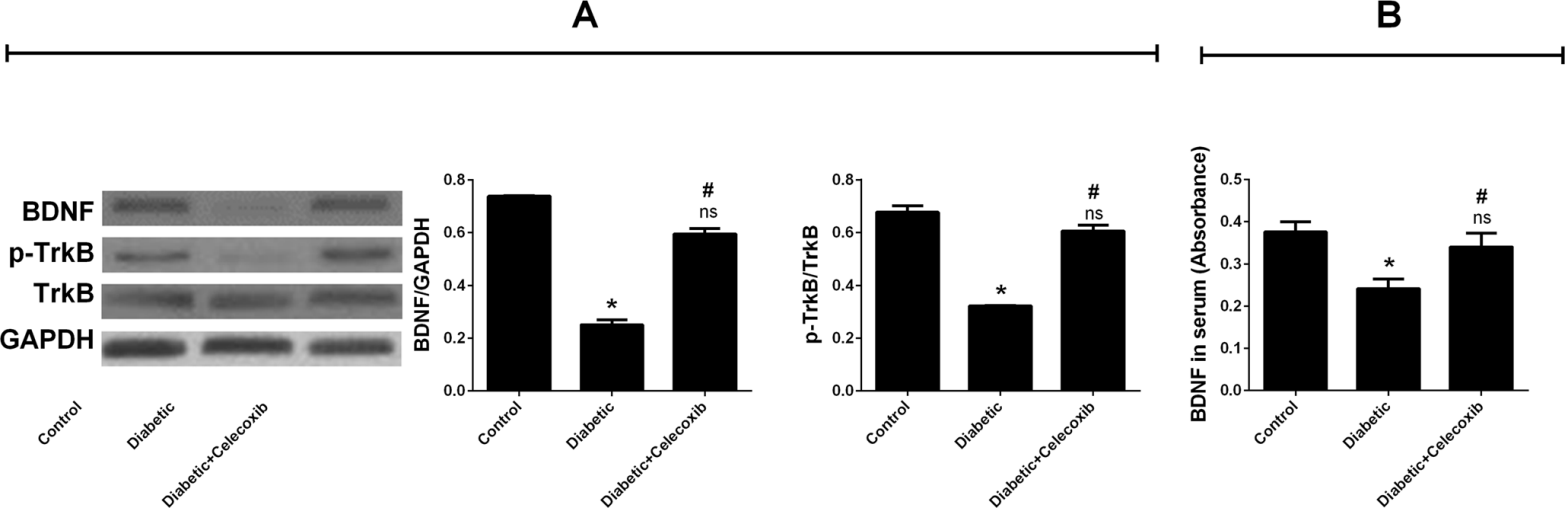
Celecoxib improves the damage in brain-derived neurotrophic factor (BDNF)-tyrosine receptor kinase B (TrkB) signaling pathway in hippocampus of the diabetic model. **a** Western blot analysis and quantification of blot density ratios of BDNF to NADPH and p-TrkB to TrkB in the three groups. **b** Concentrations of serum BDNF were detected by ELISA. Serum BDNF levels decreased in diabetic animals compared to control group, whereas celecoxib attenuated the decrease (mean ± SEM, *n* = 5 animals/group). *Asterisk* indicates that *P* < 0.05 as compared to the control group. *Number sign* suggests that *P* < 0.05 as compared to diabetic group. *ns* no significance, *BDNF* brain-derived neurotrophic factor

The BDNF-TrkB signaling pathway plays an important role in LTP and learning memory. We measured the protein levels of hippocampal BDNF and TrkB in the rat and found that BDNF level in the hippocampal homogenate was markedly downregulated in the diabetes model group (Fig. 4a). The p-TrkB expression was also downregulated in the same group. No significant difference was recorded for the TrkB expression between groups. Celecoxib treatment upregulated BDNF and TrkB phosphorylation compared to the diabetic group (Fig. 4a). In addition, serum BDNF levels decreased in diabetic animals compared to control group, whereas celecoxib attenuated the decrease (Fig. 4b) according to the results obtained from the ELISA test.

### Celecoxib Treatment Inhibited COX-2 Activity in Diabetic Rats

**Fig. 5:**
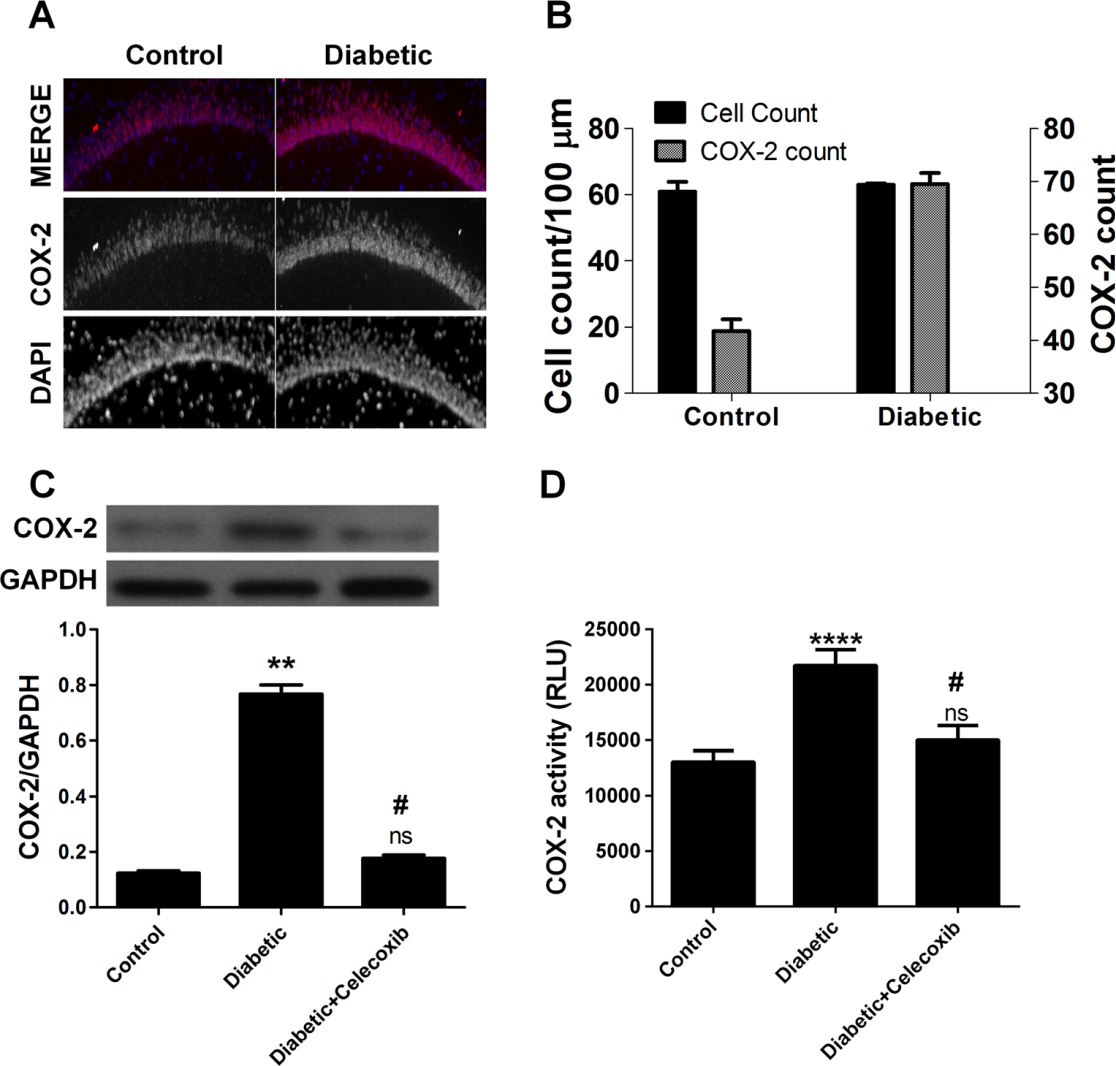
Celecoxib treatment inhibited COX-2 activity in diabetic rats. **a** Immunohistochemistry staining showed COX-2 expression in hippocampal tissue of normal control and diabetic model. **b** The number of cells per 100 μm in hippocampal tissue and the number of COX-2^+^ cells per 100 μm in hippocampal tissue of normal control and diabetic model as obtained from Immunohistochemistry staining. The number of total cells and COX2^+^ cells per 100 μm was counted by “analyzing particles” function using ImageJ software. The threshold was set at diameters of 50 to 100 μm (mean ± SEM, *n* = 16 animals/group). **c** Western blot analysis and quantification of blot density ratio of COX-2 to NADPH demonstrated that COX-2 expression in hippocampal tissue of diabetic rat was inhibited following celecoxib treatment. **d** COX-2 activity in hippocampal tissue of diabetic rat was inhibited following celecoxib treatment. Diabetic animals had increased COX-2 activity compared to control group. The increase in COX-2 activity in diabetic animals was attenuated by celecoxib treatment (mean ± SEM, *n* = 4 animals/group). *Two*, *three*, and *four asterisks* indicate that *P* < 0.01, *P* < 0.001, and *P* < 0.0001 as compared to the control group. *Number sign* indicates that *P* < 0.05 as compared to diabetic group. *ns* no significance, *COX-2* cyclooxygenase-2, *DAPI* 4′,6-diamidino-2-phenylindole, *GADPH* reduced glyceraldehyde-phosphate dehydrogenase

Immunohistochemistry staining of hippocampal for COX-2-positive cells showed that there was only faint immunoreactive staining detected in the hippocampi of rats in the control group, while sections from STZ-treated rats showed patches of COX-2-positive cells spread over the whole areas (Fig. 5a, b). Western blot demonstrated that COX-2 expression increased significantly in hippocampi of STZ-induced diabetic animals compared to normal control, but the increased COX-2 expression was inhibited by celecoxib treatment (Fig. 5c). These results demonstrated that the increase in COX-2 may be the main cause leading to memory deficit in the rat model of diabetes. Further, hippocampal tissue was harvested at day 10 after celecoxib treatment and COX-2 activity in hippocampal tissue among control, diabetic, and diabetic + celecoxib groups was detected. The COX-2 activity was significantly different among the three groups (Fig. 5d). Diabetic animals had increased COX-2 activity compared to control group (Fig. 5d). The increase in COX-2 activity in diabetic animals was attenuated by celecoxib treatment (Fig. 5d). These results indicated that celecoxib treatment improves memory deficit by inhibition of COX-2 expression in diabetic rats.

## Discussion

Mechanisms of learning and cognitive impairment in diabetes have been investigated by different studies. Diabetes-associated comorbidities were shown to affect learning and memory skills (Strachan et al. 1997). The prospective Framingham Heart Study with 1811 participants indicated that diabetes and hypertension have both independent and synergistic effects on cognitive function, particularly on learning and memory skills (Elias et al. 1997). Hypercholesterolemia also affects memory performance independently in elderly patients with type 2 diabetes (Desmond et al. 1993). In addition, the memory deficits have been attributed to various physiological mechanisms, such as dysregulation of the hypothalamic-pituitary-adrenal (HPA) axis, vascular complications, glucose metabolism, and insulin resistance (Helkala et al. 1995). The impact of diabetes on memory has been further examined by structural and functional neuroimaging. Structural brain imaging has suggested that global atrophy of the brain involve in memory impairments in diabetes. Increased loss of neurons, brain infarcts caused by obstructed blood vessels, and hyperintensities in white matter were shown to cause poor cognitive performance (Tiehuis et al. 2009). Localized atrophy in the hippocampal area was further identified as the cause of memory deficits in diabetic patients. Patients with type 2 diabetes mellitus demonstrated increased hippocampal atrophy compared to healthy controls, with verbal episodic memory domain being affected significantly (den Heijer et al. 2003). Meanwhile, functional magnetic resonance imaging (fMRI) demonstrated decreased connectivity between the hippocampus and surrounding brain structures, especially the frontal and temporal gyri (Zhou et al. 2010).

In the present study, we have demonstrated that STZ induction of diabetes led to the impairment of learning memory and hippocampal LTP. Our results showed that the celecoxib treatment contributes to improved memory function in the diabetic rats. These results suggest the supplementation of celecoxib for contribution to improving the cognitive capability in diabetic rats. The Morris water maze experiment has been established as an efficient method to assess spatial learning and memory in rodents. Memory ability, a key factor that reflects cognition, is an essential parameter for the study of cerebral functions. Our results show that celecoxib treatment attenuated the damage in the hippocampus CA1 zone in rats compared with that of the STZ-induced diabetic group, indicating that celecoxib has the ability to enhance memory capabilities, and its action is closely associated with its role in protecting the hippocampus CA1 region.

Studies in humans and rodents suggest that memory function is closely linked to the hippocampus and particularly attributed to the numbers and functional capability of nerve cells in the CA1 region. Our results show that celecoxib treatment causes a decrease in the COX-2 level and activity, implying this celecoxib-mediated action is beneficial to resume the brain function. Diabetic patients have chronic low-level inflammation, evidenced by increased proinflammatory cytokines and vascular expression of COX-2, the inducible isoform of COX (Savoia and Schiffrin 2007; Bagi et al. 2006). Activation of COX-2 has been identified in the aorta (Guo et al. 2005), mesenteric artery of diabetic rats (Retailleau et al. 2010), femoral artery of STZ-induced diabetic rats (Nacci et al. 2009), and coronary arteriole of diabetic patients (Szerafin et al. 2006). Enhanced NF-κB activity impairs vascular function by COX-2-dependent mechanisms in type 2 diabetic rats (Kassan et al. 2013). In addition, mRNA and protein levels of COX-2 were revealed to be elevated in the myocardium of STZ-induced diabetic rats and rats (Rajesh et al. 2012; Guo et al. 2007). COX-2 activity has also been indicated in autoimmune destruction of beta cells, and inhibition of COX-2 protects against the development of diabetes mellitus (Tabatabaie et al. 2000). The current study demonstrated for the first time that the expression of COX-2 was upregulated in the hippocampus of diabetic animals compared to normal control group, providing further evidence for the important role of COX-2 in the pathogenesis of diabetes, especially learning and cognitive function. The upregulation of COX-2 in hippocampus of diabetic animals might be due to direct inflammatory damage of hippocampus, given the high levels of inflammatory cytokines and molecules detected in the circulation. Glycosylation and inflammatory changes of arterioles might also cause ischemia in hippocampus of diabetic animals. The inflammatory and ischemic injury of hippocampus may cause memory deficit and impair hippocampal BDNF-TrkB signaling pathway in diabetic animals. BDNF and p-TrkB were decreased in diabetic model, suggesting that COX-2 may be associated with degradation of BDNF and p-TrkB. The degradation may be related to inflammatory injury of hippocampus caused by either high levels of inflammatory cytokines and molecules detected in the circulation or glycosylation and inflammatory changes of arterioles supplying hippocampus. BDNF/TrkB signal transduction pathway participates in enhanced learning and memory during chronic multiple stress (Li et al. 2007). Acute and chronic interference with BDNF/TrkB signaling damages LTP in the CA3 region of mouse hippocampus (Schildt et al. 2013). Decreased mRNA expression of BDNF, TrkB, and glutamic acid decarboxylase was also shown in the hippocampus of individuals with schizophrenia and mood disorders ().

Meanwhile, we revealed that COX-2 inhibitor celecoxib improves memory deficit and damage of hippocampal LTP in the diabetic model, indicating COX-2 is an essential therapeutic target for diabetes-associated memory and learning deficit. We further revealed that celecoxib reversed the damage in hippocampal BDNF-TrkB signaling pathway in a rat model of diabetes. BDNF and p-TrkB were decreased in diabetic model, which were effectively reversed by COX-2 inhibitor celecoxib. Therefore, COX-2 may be associated with degradation of BDNF and p-TrkB. The current study demonstrated celecoxib reversed the decrease in the expression of hippocampal BDNF-TrkB in a rat model of diabetes. Because BDNF/TrkB signaling pathway plays an important role in enhanced learning and memory, the restoration of protein expression of BDNF and p-TrkB by celecoxib may account for the protective role of COX-2 inhibition in diabetes-associated memory and learning deficit.

## Conclusion

We have provided novel data indicating that COX-2 and BDNF/TrkB signaling pathways are pathological and therapeutic targets for memory deficits in a rat model of diabetes. Although further research endeavor is needed to elucidate the underlying cellular and molecular signaling events, we showed that celecoxib alleviated memory deficits in diabetic rats by targeting these pathways, which constitutes promising options for treatment of memory deficits associated with diabetes in human patients.
